# High throughput exploration of process-property linkages in Al-6061 using instrumented spherical microindentation and microstructurally graded samples

**DOI:** 10.1186/s40192-016-0054-3

**Published:** 2016-06-14

**Authors:** Jordan S. Weaver, Ali Khosravani, Andrew Castillo, Surya R. Kalidindi

**Affiliations:** 1 George W. Woodruff School of Mechanical Engineering, Georgia Institute of Technology, Atlanta, GA USA; 2 Center for Integrated Nanotechnologies, Los Alamos National Laboratory, Los Alamos, NM USA

**Keywords:** Sample libraries, High throughput, Hertzian indentation, Al alloys, Aging

## Abstract

Recent spherical nanoindentation protocols have proven robust at capturing the local elastic-plastic response of polycrystalline metal samples at length scales much smaller than the grain size. In this work, we extend these protocols to length scales that include multiple grains to recover microindentation stress-strain curves. These new protocols are first established in this paper and then demonstrated for Al-6061 by comparing the measured indentation stress-strain curves with the corresponding measurements from uniaxial tension tests. More specifically, the scaling factors between the uniaxial yield strength and the indentation yield strength was determined to be about 1.9, which is significantly lower than the value of 2.8 used commonly in literature. The reasons for this difference are discussed. Second, the benefits of these new protocols in facilitating high throughput exploration of process-property relationships are demonstrated through a simple case study.

## Background

Recent advances in spherical nanoindentation protocols and data analyses have demonstrated the ability to reliably and consistently extract the mechanical response in small material volumes in the form of indentation stress-strain (ISS) curves [1–12]. The majority of these studies have focused on very small length scales (regions typically within individual grains of a polycrystal). It is important to recognize that these new protocols rely on the continuous stiffness measurement (CSM) [13–15], now readily accessible in many modern nanoindenters. Indeed, CSM facilitates a reliable estimate of the evolution of the contact radius during the entire indentation test and makes it possible to extract a meaningful indentation stress-strain curve that exhibits an initial elastic regime, an elastic-plastic transition, and a post-yield response.

**Fig. 1:**
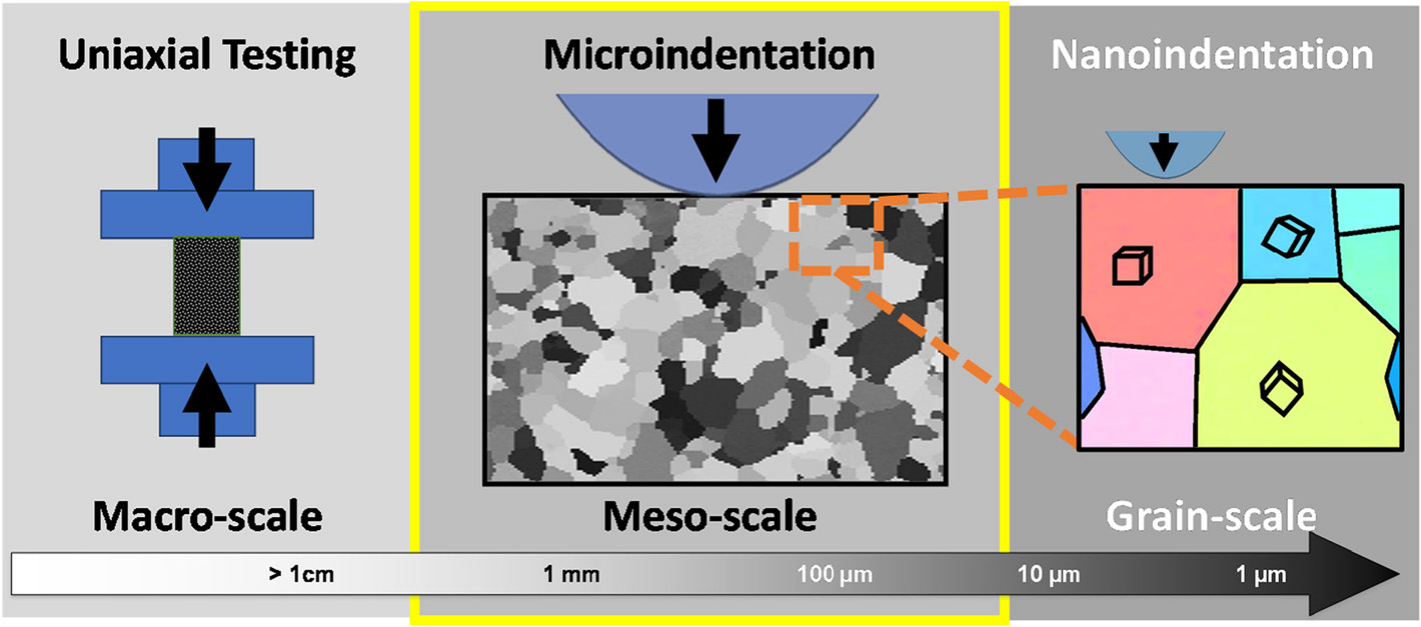
Indentation schematic of nanoindentation measurements which record the grain scale response and microindentation measurements which record a mesoscale response

However, the gap in the length scales between the nanoindentation protocols mentioned above and the standardized bulk mechanical tests [16] is too large, as illustrated in Fig. 1. There is therefore a critical need to develop validated techniques for measuring local mechanical response in polycrystalline metals at the scale of several grains (say ~10 to ~100 grains). This information is essential to produce robust physics-based connections between the grain-scale and the bulk mechanical responses. There are many potential avenues and strategies to fill this critical gap. Numerous efforts in current literature have explored small-scale testing (e.g., [17–22]). However, these methods are intensive in both effort and cost. Indentation techniques have an inherent advantage in that they can potentially result in high throughput assays, while requiring only small volumes of material.

It should be noted that the currently available nanoindenters cannot be easily reconfigured to conduct the desired microindentation tests by simply using much larger indenter tips. This is mainly because of the limits in the loads the nanoindenters can apply on the sample, and the inherent operational limits of the current CSM modules. On the other hand, none of the current microindenters allow a CSM in addition to the measurements of load and displacement.

Other than the instrumentation challenges described above, the other major challenge with indentation techniques is the correlation of properties measured in indentation with those measured in the standardized bulk tension tests. For the purpose of this paper, bulk tension and bulk compression tests are treated to produce the same uniaxial stress-strain response; this is a reasonable assumption for most metals. Numerous prior studies have explored the correlations between indentation and uniaxial measurements, as summarized by P. Zhang et al. [23]. As early as the 1950s, Tabor [24] demonstrated that the uniaxial stress-strain response could be related to hardness curves (a collection of hardness measurements at different indentation loads or indentation depths) produced using a ball indenter. Most importantly, Tabor’s study suggested the use of constraint factor of 2.8 (accounting for the higher hydrostatic pressure in the indentation tests) to relate hardness to the uniaxial yield strength. However, missing from Tabor’s experiments are the elastic loading, elastic-plastic transition, and early plastic deformation. Arguably, his hardness measurements represent effective flow stresses at relatively large plastic strains. This then means that estimating uniaxial yield strength on an engineering stress-strain curve from hardness measurements entails an extrapolation. This extrapolation introduces a large amount of uncertainty in the estimated uniaxial yield strength. In order to reduce this uncertainty, the elastic loading, elastic-plastic transition, and early plastic deformation need to be captured using highly reliable and repeatable protocols. Making matters worse is the use of sharp tip geometries (e.g., Berkovich and Vickers) which impose plastic deformation almost immediately upon loading, making it nearly impossible to recover this critical information from indentation tests. There have indeed been a few note-worthy instrumented spherical microindentation studies based on Tabor’s main idea. Although these have demonstrated the recovery of the elastic and plastic properties [25–27], critical information (the elastic-plastic transition) is still missing from test protocols and analyses. As noted earlier, this level of sophisticated analyses of indentation raw data to produce reliable and meaningful ISS curves has thus far only been possible with spherical nanoindentation [28].

The main goals of this study are twofold. First, we report on our efforts to conduct instrumented microindentation tests on a universal hardness testing machine and recover ISS curves. The details of the testing and analyses protocols are discussed. The ISS curves produced on Al-6061 are compared with the uniaxial stress-strain curves to recover the constraint factor (the ratio of the indentation yield strength to the uniaxial yield strength). Second, we demonstrate the potential of microindentation protocols developed here for rapid (high throughput) exploration of process-property linkages in Al-6061.

## Methods

A major goal of the present work is to establish a relationship between the mesoscale spherical microindentation stress-strain measurements and standardized tensile tests. Consequently, in this work, we have conducted both types of mechanical tests (i.e., tension tests and spherical microindentation tests) on selected samples so that we can critically explore the correlations between properties measured in the two different test types. As it was described earlier, the elastic-plastic transition or indentation yield strength determined using spherical indentation stress-strain protocols is a more physically relevant measurement to establish correlations with uniaxial strength. This is the first mesoscale study using protocols based on the spherical nanoindentation protocols of Kalidindi and Pathak [4, 29].

### Materials

The aluminum alloy 6061 was chosen for this study because of its industrial importance and sensitivity to thermal processing (i.e., aging). A large plate, approximately 30 × 30 × 2.5 cm thick, was acquired from Mercury Marine (Fond du Lac, WI) in the T6 condition with nominal chemical composition listed in Table 1. All metallographic specimens presented in this work were ground and polished to 0.06-μm colloidal silica with a final step of electro-polishing. Electron backscatter diffraction (EBSD) maps were produced using a Tescan Mira XMH field emission scanning electron microscope (FE-SEM) with an EDAX Hikari camera and TSL OIM Software. Grain size and orientation distribution (i.e., texture) were extracted from these scans.

**Table 1:** Nominal chemical composition of as-received Al-6061 measured by inductively coupled plasma (ICP) on 1 cm^3^

Element	Al	Cr	Cu	Fe	Mg	Mn	Si	Ti	Zn
Min %		0.04	0.15		0.8		0.40		
Max %		0.35	0.40	0.7	1.2	0.15	0.80	0.15	0.25
Actual %	Remainder	0.20	0.30	0.5	1.0	0.12	0.46	<0.01	0.10

### Mechanical tests

**Fig. 2:**
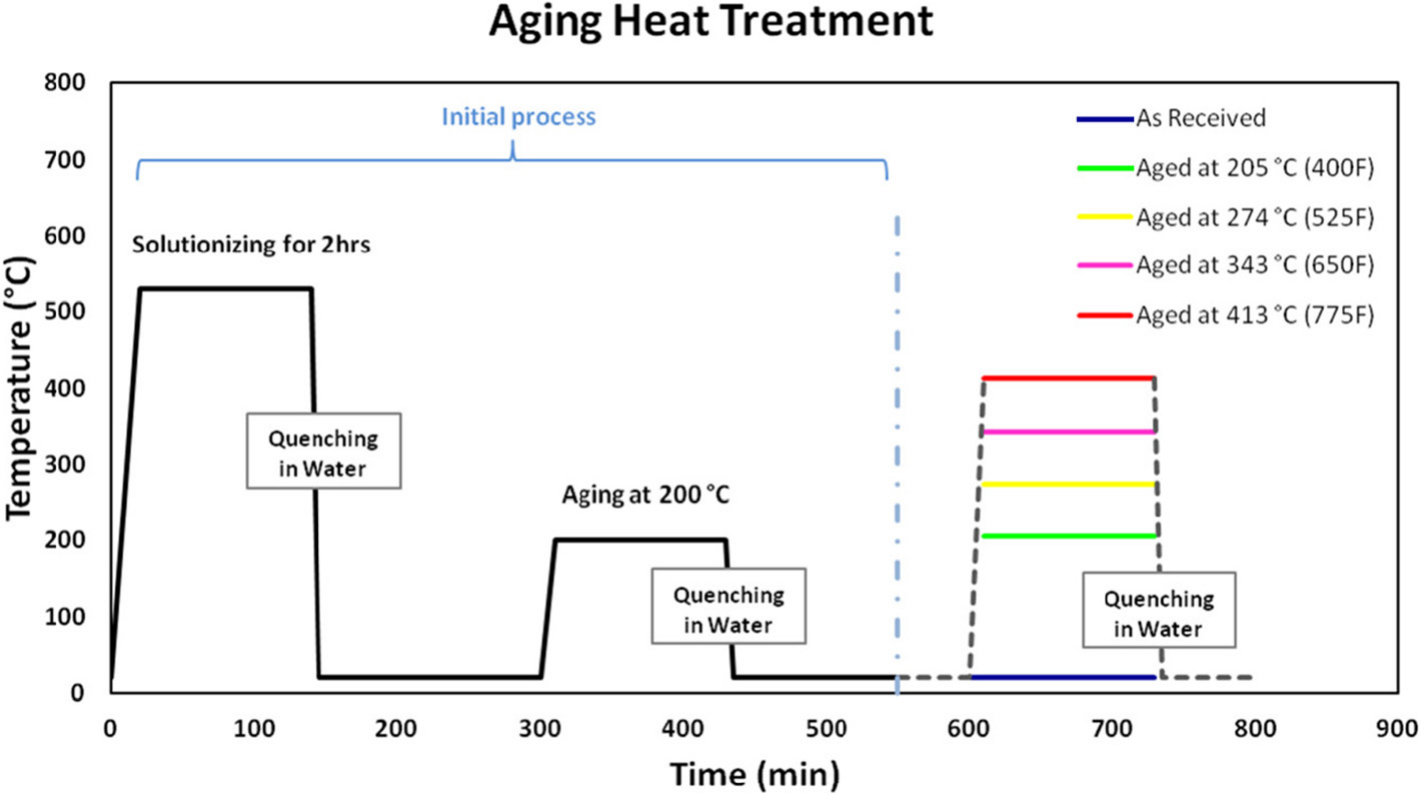
Thermal heat treatment process for Al-6061T6 condition of the as-received material and the aged samples at different temperatures

Tensile testing was performed on an Instron load frame with a 900-kN capacity load cell. Tests were run in accordance with ASTM Standard E8-13a with a constant cross head speed to produce a strain rate of 0.005 s^−1^ [16]. Specimens had a diameter and gage length of 0.635 and 2.54 cm, respectively. The gage length displacement was measured with a 2.54-cm clip gage. Tensile samples were excised from the as-received (AR) material along the rolling direction (RD) of the plate. Tests were also conducted in the TD direction as well as at 45° to RD direction. Since the measurements showed very little anisotropy, the measurements in these other directions have not been included in this paper. Following protocols employed in conventional material development efforts, additional tensile samples in the RD direction were aged for 2 h at different temperatures to explore the effect of aging heat treatments (see Fig. 2) using a salt bath furnace, followed by quenching in water. Aged material was kept in a freezer between heat treating and characterization to avoid any natural aging.

**Fig. 3:**
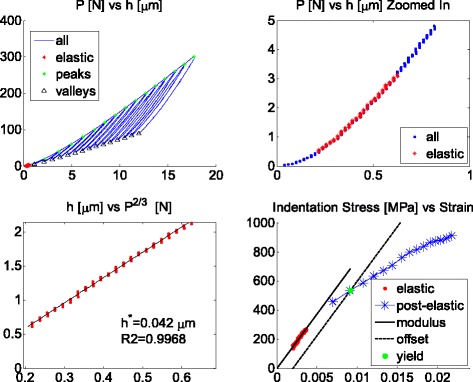
Example microindentation load-displacement curve, analysis procedure, and stress-strain curve. The *red data* corresponds to the initial elastic segment used to determine the effective modulus. The linear regression of this data, *bottom left*, also determines the displacement zero point correction. In this example, no load correction was applied. Each unload is analyzed using the same linear regression to determine the contact radius

Indentation testing was performed on a Zwick-Roell Z2.5 Zwicki Hardness Tester with the indentation axis parallel to the ND plane normal. The opposing surface to the test surface was ground flat and parallel. Specimens were mounted to a precision ground hardened steel plate with a thin layer of adhesive. Indentation tests were run with a constant crosshead speed of 0.1 mm/min with incremental unloading (50–30 % of the peak force) and reloading cycles until the specified number of cycles was reached, as shown in Fig. 3. These unloading cycles are essential to the recovery of the ISS curves, as will be discussed later. A spherical tungsten-carbide tip was used for all tests, which had a nominal composition of 94 wt.% WC and 6 wt.% Co and a radius of 6.35 mm.

### Microindentation data analyses

The general method followed for converting indentation load-displacement data into indentation stress-strain curves can be found in references [4, 29]. It is emphasized that this is the first use of these protocols to study polycrystalline material volumes in the primary indentation zone. For microindentation testing equipment used in this work, there is no CSM as noted earlier, this is only available in most modern nanoindenters [30]. As demonstrated in prior work [4, 29], the measurement of unloading (elastic) stiffness is central to a reliable estimation of contact radius needed in the computation of indentation stress and indentation strain measures. Consequently, in the present work, we rely on superimposing intermittent unloading-loading cycles on the desired loading history. Note that each unload results in the estimation of one data point on the ISS curve (i.e., one value of indentation stress and indentation strain; [29]). Several challenges arise in using these protocols for microindentation. First, it is difficult to capture the initial elastic loading with an instrumented microindenter. Nanoindentation systems have adequate load and depth sensing resolutions needed to capture the initial elastic loading; however, instrumented microindenters are not well designed for capturing the initial elastic loading segment. The higher loads required generally for mesoscale measurements (10^2^–10^3^ N) significantly reduce the load resolution during the early stages of loading, while the best displacement resolution currently available is around 20 nm. Second, the higher loads present a significant challenge in the sample alignment and mounting. For a successful test, the top and back surfaces of the sample must be polished parallel to each other and set directly on a rigid surface (hardened steel plate or directly on the stage); otherwise, misalignment, sample rotation, or compliance issues will produce erroneous results. Third, the combination of higher loads and less rigid indenter tips means that the elastic displacement of the tip may be significant. For example, the elastic displacement of the tip (usually diamond with a modulus >1000 GPa) is typically negligible in nanoindentation, especially with the low loads (<500 mN). The same assumption is likely to be a poor assumption for tungsten carbide tips (around half the modulus of diamond) at the higher loads seen in microindentation. Therefore, a suitable correction is needed in the analysis to account for the tip displacement based on the elastic properties of the tip material. Below, we briefly review the main details of the indentation data analyses protocols used in this study.

Hertz theory (Eqs. (1)-(4)) describes the frictionless, elastic contact between two isotropic, homogenous bodies with parabolic surfaces [31].

1 $$ P=\frac{4}{3}{E}_{\mathrm{e}\mathrm{ff}}{R}_{\mathrm{e}\mathrm{ff}}^{1/2}{h}_{\mathrm{e}}^{3/2} $$

2 $$ a=\sqrt{R_{\mathrm{e}\mathrm{ff}}{h}_{\mathrm{e}}} $$

3 $$ \frac{1}{E_{\mathrm{eff}}}=\frac{1-{v}_{\mathrm{i}}^2}{E_{\mathrm{i}}}+\frac{1-{v}_{\mathrm{s}}^2}{E_{\mathrm{s}}} $$

4 $$ \frac{1}{R_{\mathrm{eff}}}=\frac{1}{R_{\mathrm{i}}}+\frac{1}{R_{\mathrm{s}}} $$

In the above equations, *P* and *h*_e_ denote the indentation load and displacement, respectively. *R*_eff_ and *E*_eff_ denote the effective radius and modulus of the combined indenter-sample system, while *a* denotes the contact radius. The subscripts i and s denote that the variables are associated with the indenter and sample, respectively.

During the early stage of loading on a flat sample surface, prior to any permanent deformation, the effective radius is equal to the indenter radius (i.e., *R*_eff_ = *R*_i_). In this initial elastic regime, it is relatively easy to extract a value of the effective modulus, *E*_eff_ using Eq. (1) and standard regression techniques. The sample Young’s Modulus, *E*_s_, can then be determined from Eq. (3), provided the sample Poisson ratio, *v*_s_, and the indenter elastic properties are known. It is important to note that our treatment of the sample as elastically isotropic (reasonable for the weak texture and very little anisotropy observed in tensile tests) is not a limitation of the analysis. Many authors have shown that the Eq. (1) extends to elastically anisotropic materials with only slight modifications to Eq. (3) [32–38]. The contact area becomes elliptical instead of circular; however, the error associated with treating it as circular is very small [34]. The contact radius can be interpreted as an effective contact radius for elastically anisotropic materials.

In nanoindentation tests, the identification of the elastic loading segment involves determining the zero-point corrections for both load and displacement [4, 29]. In microindentation, the indentation stress-strain curve is far less sensitive to surface and tip disparities because of the large tip radii, and in many cases, there is no need for load correction (see Fig. 3). The analysis in this study systematically examined different load corrections and their corresponding displacement corrections following the approach described in earlier work [29]. Based on this exploration, it was decided to select the optimal load correction as the one that minimizes the log of the average absolute residual of the linear regression fit for the elastic segment (prior to any detected residual deformation in the sample); the displacement correction is also automatically identified in this process.

After plasticity occurs, the effective radius is unknown, and the total displacement is now the sum of the elastic displacement and the permanent displacement or residual height, *h*_r_. However, unloading is primarily elastic, and therefore the contact radius, *a*, can be determined by applying Hertz’s equations to the unloading data using standard regression techniques and the following equations:

5 $$ h=k{P}^{\frac{2}{3}}+{h}_{\mathrm{r}} $$

6 $$ k={\left(\frac{3}{4}\right)}^{\frac{2}{3}}{E}_{\mathrm{eff}}^{-\frac{2}{3}}{R}_{\mathrm{eff}}^{-\frac{1}{3}} $$

In Eq. (6), *R*_eff_ is the only unknown, because *E*_eff_ was already determined from the initial loading data and is assumed to be the same even after the sample experiences plastic deformation. This assumption is reasonable because the effective (average) plastic deformation in the indentation zone is quite small in these experiments. Unloading data between 95 and 50 % of the peak load was used for each unload in this analysis. The contact radius at the point of unloading is determined from Eq. (2). After the contact radius is determined, indentation stress and strain are calculated using the following set of equations, where the subscript max represents the peak load and displacement for each unload.

7 $$ {\sigma}_{\mathrm{ind}}=\frac{P_{\max }}{\pi {a}^2} $$

8 $$ {\varepsilon}_{\mathrm{ind}}=\frac{4}{3\pi}\frac{h_{\mathrm{s}, \max }}{a} $$

9 $$ {h}_{\mathrm{s}}=h-{h}_i $$

10 $$ {h}_{\mathrm{i}}=\frac{3\left(1-{v}_{\mathrm{i}}^2\right)P}{4{E}_{\mathrm{i}}a} $$

In the above set of protocols, care was afforded to subtract the elastic displacement of the indenter, *h*_i_, in order to use only the displacement of the sample, *h*_s_, in the computation of the indentation strain in the sample, *ε*_ind_. This is accomplished using Eqs. (9) and (10), where the elastic displacement of the indenter, *h*_i_, is calculated using the Hertz’s theory (for the displacement of the indenter tip pressed into a rigid flat surface in the absence of a sample). We assume that this is a good approximation for the indenter displacement during our tests. It can be seen from Eq. (10) that the correction will be higher for indenter tips with lower moduli. In this study, a Young’s modulus and Poisson ratio of 640 GPa and 0.21 were used for the indenter, based on values reported in literature for tungsten carbide [39]. In this work, the sample modulus was computed assuming a Poisson ratio of 0.3, and the determination of indentation yield strength was made using a 0.2 % indentation plastic strain offset on the indentation stress-strain curve using an indentation modulus of $\frac{E_{\mathrm{s}}}{1-{v}_{\mathrm{s}}^2}$ (see Fig. 3).

## Results and Discussion

### Comparison of microindentation and uniaxial responses

**Fig. 4:**
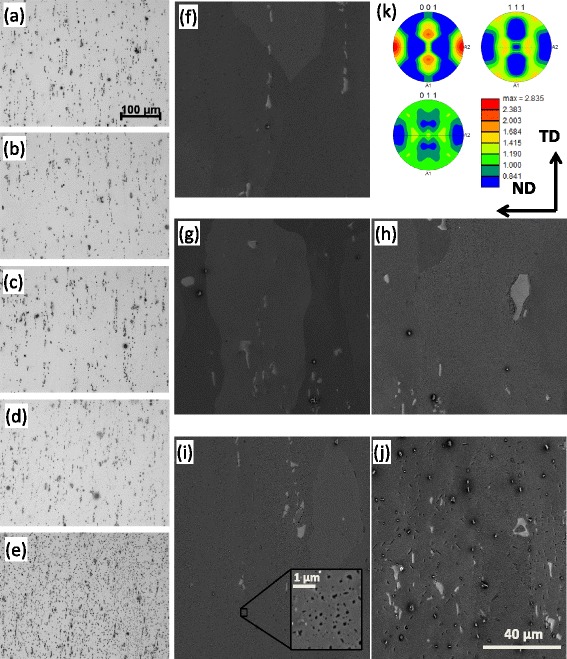
Optical and SEM-BSE micrographs of the AR material (**a**, **f**) and different aged samples: 204 °C (**b**, **g**), 274 °C (**c**, **h**), 343 °C (**d**, **i**), and 413 °C (**e**, **j**), respectively. The texture of the AR material is shown in **k**. All images are taken from the RD plane. The average grain diameter measured on an area containing ~450 grains using EDAX OIM Analysis software was 59 μm, and the average aspect ratio was 0.40

**Fig. 5:**
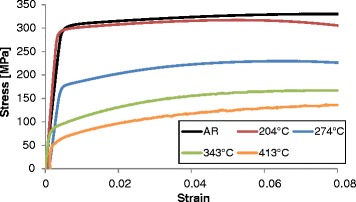
Tensile stress-strain curves for the as-received material (AR) and different aging temperatures

Optical and SEM-BSE micrographs of the AR material and heat-treated samples can be seen in Fig. 4, which shows an increase in large precipitates with increasing aging temperature. The grain size distribution and texture were not affected by aging temperature. Tensile testing revealed decreasing tensile strength with increasing aging temperature (see Fig. 5). The strength of Al-6061 is largely controlled by the amount and types of Mg_2_Si precipitates [40–43]. A detailed characterization and quantification of the types and amounts of precipitates present in the samples subjected to various heat treatments requires transmission electron microscopy (TEM) which is outside the scope of the present study. However, it is clear from the micrographs shown in Fig. 4 that the aging treatment causes an increase in large precipitates, likely *β* precipitates which do not directly affect the strength, meaning there is a reduction in *β* ^”^ precipitates, which have the most influence on strength. This is consistent with the observed trends in the tensile test results shown in Fig. 5.

**Fig. 6:**
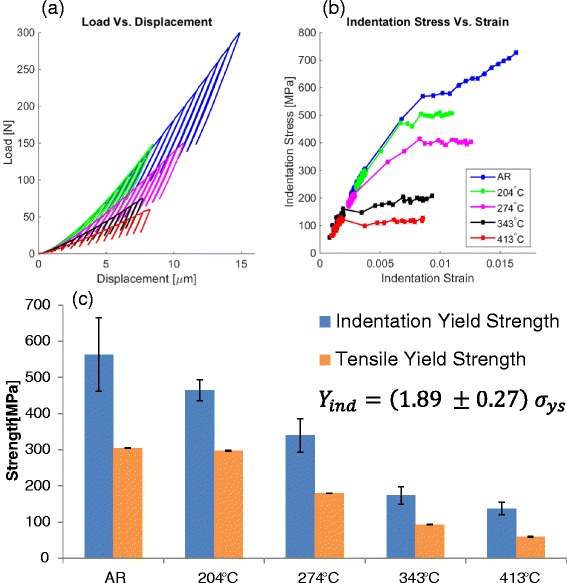
Correlations between microindentation and uniaxial experiments: **a** load-displacement curves measured with a 6.35-mm tip radius for five different process conditions: As-received (AR), aged 2 h at 204, 274, 343, and 413 °C, **b** corresponding indentation stress-strain (ISS) curves, and **c** the correlation between indentation and uniaxial yield strength. Indentation yield strength and tensile yield strength are averages with error bars representing ± one standard deviation for approximately 15 and 6 tests, respectively, for each condition. The average ratio is calculated based on all five conditions

Indentation stress-strain measurements on the samples subjected to the same aging treatments are presented in Fig. 6, and they serve to make direct comparisons with the tensile tests on bulk samples reported in Fig. 5. Indeed, the indentation measurements reveal the same trends seen in the bulk tensile samples (i.e., decreasing strength with aging temperature). Furthermore, the ratio between the 0.2 % offset indentation yield strength and the 0.2 % offset tensile yield strength was observed to be about 1.9, with a standard deviation of 0.3. This ratio accounts for the fact that the hydrostatic stress component is significantly higher in the indentation test conditions, compared to the uniaxial stress conditions.

**Fig. 7:**
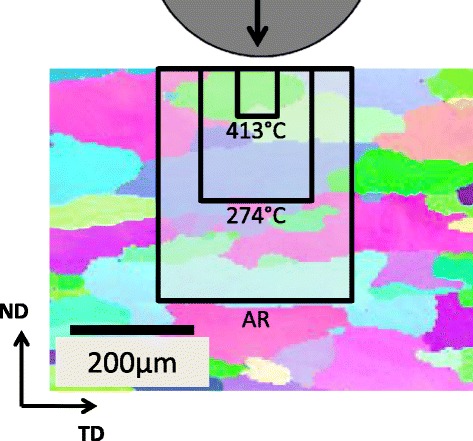
Overlay of primary indentation zone (2*a* × 2.4*a*) at yield on an EBSD grain map. The average contact radius at yield for each condition was 156 μm (AR), 158 μm (204 °C), 103 μm (274 °C), 92 μm (343 °C), and 31 μm (413 °C). The indentation direction is parallel to the normal direction (ND)

The length scale of the indentation zone in our experiments was determined based on the concept of a primary indentation zone defined as a cylindrical volume of radius *a* and height 2.4*a* [4]. The variation in the zone size with respect to the microstructure for different individual heat treatments can be seen in Fig. 7. This zone size is an estimate of the probed material. With the average contact radius and the average grain size (54 μm), one can estimate the number of grains inside the primary indentation zone (i.e., the volume of the primary indentation zone divided by the volume of the average grain assumed to be a sphere). This information is listed in Table 2. At the high end (AR and 204 °C samples), the number of grains is approximately 360, and at the low end (413 °C sample), the number of grains is approximately 3. In all cases, there is a high probability of grain boundaries present in the indentation zone at yield. The difference between samples is primarily the number of grains in the polycrystalline volume. Despite the relatively small number of grains for the sample aged at 413 °C, the indentation yield strength correlates well with the bulk uniaxial yield strength. Had the primary indentation zone been even smaller (i.e., a smaller indenter size), the mechanical response would likely be dominated by individual grain orientations at the indentation site and not represent a bulk strength measurement. The protocols for extracting hardening rates from indentation stress-strain curves are being established at this time. However, the representative indentation stress-strain curves in Fig. 6 qualitatively show there is no trend in the indentation hardening rate with aging temperature.

**Table 2:** Primary indentation zone size (PIZ) at yield and the estimated number of grains in the PIZ

Condition	Contact radius at yield [μm]	PIZ volume [10^3^ mm^3^] (*πa* ^2^ × 2.4*a*)	Estimated no. of grains $No.=1.8\;{a}^3/{\mathrm{R}}_G^3$
AR	156 ± 16	28.7	348
204 °C	158 ± 12	29.6	359
274 °C	103 ± 35	8.2	99
343 °C	92 ± 17	6.0	72
413 °C	31 ± 3	0.2	3

### Relationships between indentation and uniaxial measurements

Literature reports a value of around 2.8 for the indentation constraint factor defined as the ratio of hardness or mean pressure to uniaxial flow stress [1, 3, 5, 24, 44–52]. These studies are primarily inspired by Tabor’s initial experiments conducted on copper and steel for which he identified a ratio of 2.8. In Tabor’s experiments, the contact radius (actually the projected contact area) was determined from residual indents after complete unloading. It should be recognized that the protocols described and employed in the present work follow the definitions arising from Hertz’s theory, where the contact radius and the corresponding projected contact area are both defined in the fully loaded condition. Furthermore, the measurements Tabor made required a significant amount of plastic deformation in order to make a residual indent, whereas this study quantified the 0.2 % offset indentation yield strength, which is well below the typical plastic strains seen in hardness measurements. The contact radius at peak load should be expected to be greater than the contact radius measured on residual indents because of the elastic recovery; this difference will be significant in the early portion of the stress-strain curve (note that for a purely elastic indentation there is no residual indentation). As such, it is very reasonable that the constraint factor obtained using the protocols presented here (~1.9) is significantly lower than the value reported by Tabor.

Further assessing the literature that supports a constraint factor of 2.8, it is noted that some researchers [1, 3, 5, 52] have indeed measured the contact radius in the fully loaded condition [2, 5, 53], which appears to negate the arguments made earlier and adds significant confusion. Our interpretation and rationale is as follows. First, the agreement on the constraint factor for protocols which measure the contact radius in the fully loaded condition and from residual indents is largely based on the material response after significant plastic deformation has occurred at the indentation site (i.e., a fully plastic zone is established at the indentation site). At such large plastic strains, the difference between the contact radius in the fully loaded condition and from residual indents, as Tabor used, is significantly less than during the early stages of plasticity. Consequently, experiments in this regime using both definitions of contact radius provide similar constraint factors. Second, the protocols used to estimate the contact radius in the fully loaded condition [2, 5, 53] in prior studies are not the same as those used in this work [4, 29]. Donohue et al. [54] critically evaluated these differences using a finite element model as a surrogate for the indentation experiment. These authors found that the protocols used in literature estimate the actual contact radius while the protocols used in this work estimate the Hertzian contact radius—one that is consistent with Hertz’s theory. Additionally, the Hertzian contact radius was found to be larger than the actual contact radius once plasticity initiates (even at small plastic strains). Once more, it becomes apparent that it is very reasonable that the constraint factor presented here (~1.9) is significantly lower than the value reported by Tabor and others (around 2.8).

Models and finite element simulations also provide additional insights regarding the constraint factor for the protocols used in this work. For example, a simple application of the Mises yield criterion to the elastic stress fields predicted by Hertz theory suggests that plasticity initiates inside the indentation zone around a value of 1.1*σ*_*y*_, where *σ*_*y*_ represents the uniaxial yield strength of the sample. However, this value reflects only the initiation of plasticity in the indentation zone and is very difficult to discern or validate in the actual experiment [5]. In addition to exploring differences in estimating the contact radius in the fully loaded condition, Donohue et al. [54] modeled the point of deviation from the linear elastic regime for an isotropic elastic-plastic material with protocols that are very consistent with the protocols described in this paper. This study reported a constraint value of about 1.3 at the point of deviation from the linear elastic regime in the indentation stress-strain curve [54]. This point is also very difficult to establish in the experiments as it is very sensitive to test parameters, such as the loading rate and data acquisition rates. Indeed, this is also the reason why one generally adopts an offset definition of the yield point (instead of deviation from linearity) even in the conventional uniaxial tension and compression tests on bulk samples. Very recent finite element studies [55] have identified a value of 2.2 for the constraint factor in indentation tests for points corresponding to an offset definition of the indentation yield point. It should be noted that this value corresponds to isotropic elastic-plastic response of the sample material obeying the J_2_ flow theories. Keeping in mind that the real material behavior in our tests is likely to deviate somewhat from this idealized material law, it is remarkable that our experiments have indicated an average value of 1.9 with a standard deviation of 0.3.

It is not expected that the constraint factor of 1.9 determined for the Al-6061 studied here will be constant across all material systems (e.g., it will depend on various attributes of the material mesostructure). Furthermore, some uncertainty in relating the indentation strength to the uniaxial strength is unavoidable because of the heterogeneity inherent to the indentation measurement. In other words, the deformation processes in indentation and uniaxial testing are inherently different. However, the microindentation protocols demonstrated here arguably reduce this uncertainty by capturing the elastic-plastic transition (indentation yield strength) as opposed to hardness. Just to be clear, we are not suggesting that the constraint factor of 2.8 reported in literature is incorrect. We are suggesting that the protocol for relating indentation measurements to uniaxial yield strength can be vastly improved using the microindentation stress-strain protocols and indentation yield strength measurement demonstrated in this study which inevitably leads to a different constraint factor (1.9). For example, it is expected that the indentation yield strength will show significantly less dependence on the material hardening behavior because the plastic deformation at the indentation yield point is minimal compared to hardness measurements. The ratio of 1.9 is likely to extend to other material systems which have similar anisotropies (i.e., cubic materials with weak textures). Further improvements to microindentation testing and analyses will allow for even greater confidence in high throughput screening methods for new structural alloys.

### Application: high throughput exploration of process-property linkages

The Materials Genome Initiative (MGI) has set forth the goal of cutting the materials discovery, development, and deployment time in half while simultaneously reducing the cost [56]. An important strategy for the practical realization of these goals comes from the development and adoption of novel protocols for extracting critically needed materials information or knowledge in a high throughput manner. As a prime example, the functional and biological materials communities have successfully adopted combinatorial approaches for creating the requisite knowledge systems in their fields [57–61]. Such combinatorial approaches generally involve synthesis methods/protocols for generating materials or sample libraries that contain small volumes of a substantially large variety of materials corresponding to different chemistries and/or process histories. Once the materials/sample library is produced, a high throughput screening method is generally applied to evaluate the material performance of interest. It is important that the property/performance characterization tools and techniques are able to reliably assess the property of interest from the small material volumes available in the sample library. These high throughput elements are critical to the successful realization of the acceleration in materials innovation envisioned by MGI.

In structural materials development, there have been numerous efforts aimed at high throughput explorations [62–71]. A number of these prior efforts have either employed standard tensile testing [62] (demands sufficiently large amounts of material samples produced with excellent control of processing history in the entire sample volume) or grossly simplified hardness measures [72] (these should largely be treated as qualitative measures as they generally correspond to a finite amount of plastic strain that is not maintained constant for the different samples and materials tested). A relatively small number of studies have actually employed nanoindentation [65, 66, 69–71]. However, as discussed in prior work, the protocols used in the analyses of these datasets do not produce consistent and reliable values. Furthermore, the nanoindentation measurements largely reflect grain-scale measurements that cannot be easily transformed to bulk (polycrystalline) values. The spherical microindentation stress-strain protocols developed and presented earlier are ideally suited for the task at hand.

We undertake a simple case study to demonstrate the use of the protocols developed and presented earlier on a microstructurally graded sample to extract processing-property relationships. This set of experiments focused on the careful design of a sample library with different thermal processes (high throughput sample prototyping) that is mechanically evaluated with spherical microindentation stress-strain measurement protocols. The library results are compared against individual samples processed through traditional methods in order to validate the high throughput protocols.

**Fig. 8:**
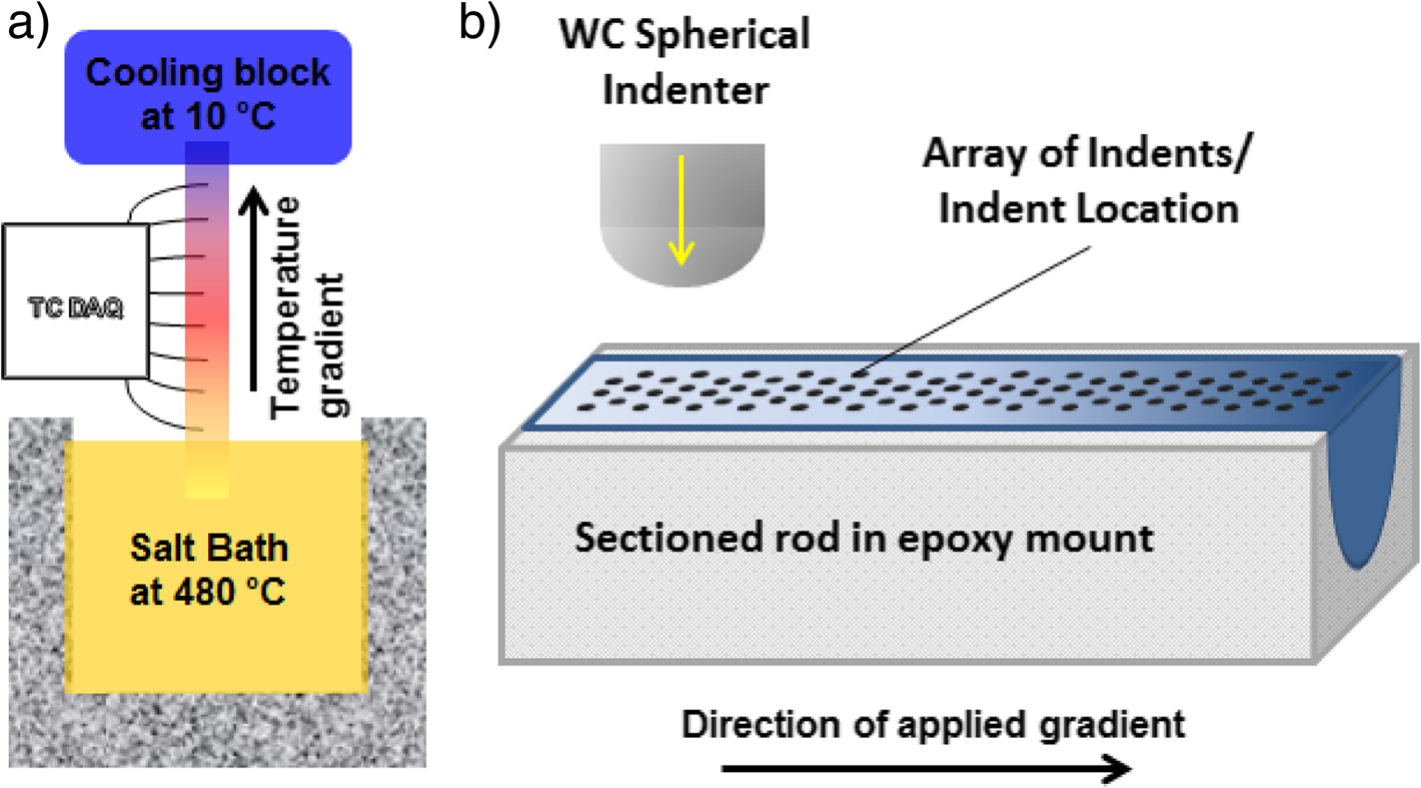
High throughput schematics: **a** custom setup for producing a sample library of different aging temperatures and **b** mechanical screening of the sample library through spherical microindentation. The spacing and size of indents is not drawn to scale

For high throughput prototyping of samples, a custom setup was designed and built to produce a single bulk sample that can serve as a sample library (Fig. 8). This setup was designed to explore the full range of aging temperatures shown in Fig. 2 in a single sample. The sample, a cylindrical rod of 1.5 cm diameter and 18.7 cm length oriented along the plate’s rolling direction, was suspended with one end in molten salt held at 480 °C, and the other end screwed into a 7.62 cm × 7.62 cm × 5.08 cm aluminum block that was cooled continuously using a chiller and a 50/50 mixture of ethylene glycol and water maintained at 10 °C. On the section of the sample that was above the molten salt, eight small, equally spaced, holes were drilled to the center of the rod to place thermocouple sensors (K-type) to measure the local temperature histories. The sample was also insulated to minimize heat loss. The sample was aged 2 h in the setup described above and water quenched. The sample was kept in a freezer between heat treating and preparation for microindentation. The sample was sectioned along the rolling direction and metallographically prepared for indentation as shown in Fig. 8. Each column (perpendicular to the applied gradient) in the array of indentation tests represents one process in the sample library. There was 3 months between the first set of experiments and the second set of experiments for which the sample was at room temperature in a low humidity environment. There was no difference observed in the indentation responses between the two sets of experiments, so we assume there was no natural aging.

**Fig. 9:**
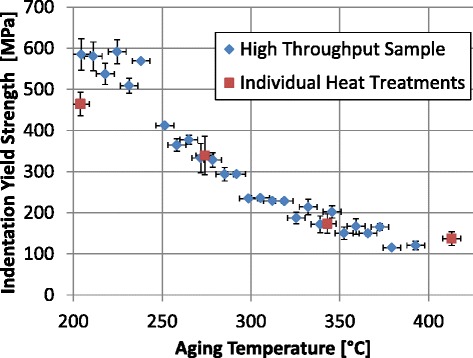
High throughput indentation experiments. Indentation measurements on the high throughput sample are in *blue*. Out of 84 tests, 76 were successfully analyzed. Each data point is the average of typically 3 tests performed at the same aging temperature (position along the bar). Error bars in the *y*-axis are ±one standard deviation. Error bars on the *x*-axis are estimated at ±5 °C. For comparison, indentation yield strength measurements for the individual heat treatments are also plotted in *red*. The error bars for these points are ±one standard deviation

For the combinatorial sample, a temperature range of ~200 to 400 °C with a linear gradient was achieved in the section monitored by thermocouples shown schematically in Fig. 8. This temperature range was chosen to cover the aging temperatures explored for individual heat treatments. Microindentation tests (84 in total) were performed in the sample section monitored by thermocouples with a uniform grid (3 rows, 28 columns with 2.5 mm spacing between columns and 2–3 mm spacing between rows). This spacing, an indent center to center spacing of six times the residual indent radius, is the accepted convention [73]. It may be possible to further reduce the spacing through experiments and simulations to develop a less conservative criterion for the minimum allowable spacing. The average Young’s modulus estimated from the indentation measurements was 69.7 GPa, with a standard deviation of 3.0 GPa and a range of 62.6 to 76.7 GPa. This compares well to the average Young’s modulus measured from all RD tensile tests which was 69.6 ± 7.6 GPa. These results provide partial validation of the indentation protocols developed and employed in this work. The position and temperature of the thermocouples were used to estimate the aging temperature at each indentation site. Indentation yield strength for each test is plotted against aging temperature in Fig. 9 which shows decreasing strength with increasing aging temperature. Indentation yield strength measurements on the combinatorial sample are generally consistent with measurements on individually heat-treated samples (Fig. 9). In one case, measurements made at 204 °C, the indentation yield strength did not agree between the individually heat-treated sample and the corresponding high throughput sample measurement. Although the reason for this discrepancy is not clear, we believe that the error might actually be in the measurement on the individually heat-treated sample at 204 °C. Note that the trends in the values measured on the high throughput sample are fairly smooth and consistent among themselves. This assumption is supported by the fact that the tensile yield strength only decreased 2 % from the AR material to the 204 °C condition, which is well captured by the measurements on the high throughput bar. Overall, it is clear that the indentation measurements on the high throughput sample can be used reliably in screening of process parameters.

In addition to the grain size relative to the primary indentation zone, there is another length scale which must be considered in the high throughput sample, which arises due to the processing temperature gradient imposed on the sample. As a result of this imposed gradient, there will be a temperature difference across the primary indentation zone. Evaluating the temperature difference across the primary zone in Fig. 7 shows there is less than a 1 °C difference across the zone, which we believe will have a negligible effect on the response measured in our experiments. This means that the individual indentation measurements on the high throughput sample should be equivalent to doing indents on individually heat-treated samples at the same temperature. Indeed, the comparison between indentation measurements on individual heat treatments and the high throughput sample shown in Fig. 9 supports this assumption.

As other researchers have demonstrated, indentation combined with a microstructurally graded sample can significantly reduce the time and effort needed to reveal materials information. The high throughput application in this study demonstrates this benefit. The reduction in time and effort was achieved by eliminating (i) the need for processing many batches of material at different temperatures, (ii) machining and testing many tensile specimens for each condition, and (iii) preparing individual samples for metallography for each process condition. In the process-property measurement protocol outlined in this paper, a single specimen was processed and metallographically prepared for both structure and mechanical characterization. The total test time for the combinatorial sample was about 2 days and could be further optimized for high throughput. The coupling of combinatorial processing and indentation stress-strain protocols has the potential to drastically reduce the time, material, and effort to improved structural alloys. The structure-processing-property space can be explored quickly to make decisions about what materials and processes are worth further exploration and more effort intensive characterization.

A critical step in the protocol developed in this study is the appropriate choice of the indenter size (radius). The appropriate choice is one that creates a sufficiently large primary indentation zone (enough to be considered polycrystalline or the equivalent of multiple microstructural features for other materials). Secondly, the indenter size should be considered concurrently with the design of a processing gradient. The most challenging case would be a sharp gradient that makes it hard to reliably measure local properties. Choosing an indenter size too large for a given gradient would create the same problem. Finally, the indenter sizes appropriate for this study required loads well beyond that of typical instrumented nano and microindentation systems. It took 400 N to recover the indentation stress-strain curve which is 40× the limit of the high load option on an XP head, Agilent G200 Nanoindenter. It is therefore clear that some effort needs to be expended in selecting the appropriate combination of the indenter size and the indentation system in designing the experiments using the protocols described in this work.

## Conclusions

Spherical indentation stress-strain protocols have been extended to an instrumented microhardness tester capable of testing larger volumes of material (polycrystalline volumes) using larger tip radii and higher loads. This capability produced two major findings:

1. This study has produced new experimental correlations between indentation and uniaxial yield strengths using a new set of protocols to improve the efficacy of such endeavors. Indentation yield strength defined using a 0.2 % strain offset on the indentation stress-strain curve was correlated to tensile yield strength for different aging temperatures on Al-6061 by a constant value of approximately 1.9 (*Y*_ind_ ≈ 1.9*σ*_*y*_), which is in good agreement with FEM simulations reported in literature for an isotropic material. This allows spherical indentation stress-strain protocols to be used as a reliable, high throughput mechanical characterization tool to quickly determine uniaxial yield strength.
2. Microindentation stress-strain curves can be inserted in high-throughput protocols for a more robust mechanical characterization of sample libraries. In this study, a combinatorial synthesis approach was used to make a single sample with many sample volumes subjected to different aging temperatures. The temperature gradient was small enough compared to the volume of material tested at each indentation site that each test could be treated as a measurement corresponding to a single aging temperature. The high throughput synthesis and mechanical characterization protocol reduced significantly the material, time, and effort needed to recover strength and aging temperature trends in Al-6061. This protocol can be applied to other thermo-mechanical processes to rapidly explore process-property trends and discover new/improved materials at a fraction of the time and cost with higher fidelity than traditional hardness and modulus measurements.

## Availability of data and materials

All aspects of the data are available upon request. The mechanical property measurements (microindentation and tensile) will be added to the NIST repository under the ASM Structural Materials Data Demonstration Project https://doi.org/materialsdata.nist.gov/dspace/xmlui/handle/11256/419

## Abbreviations

ISS, indentation stress-strain; CSM, continuous stiffness measurement; wt.%, weight percent; AR, as-received condition; PIZ, primary indentation zone; *P*, load; *h*, displacement; *E*_eff_, effective modulus; *R*_eff_, effective radius of curvature; *h*_e_, elastic displacement; *h*_r_, residual height or displacement; *E*_i_, *v*_i_, indenter Young’s modulus and Poisson’s ratio; *E*_s_, *v*_s_, sample Young’s modulus and Poisson’s ratio; *h*_s_, sample displacement accounting for elastic displacement of the indenter tip; *h*_i_, estimated elastic displacement of the indenter tip; *a*, contact radius; *R*_i_, indenter radius; *R*_s_, sample radius of curvature; *E*_ind_, indentation modulus; *σ*_ind_, indentation stress; *ε*_ind_, indentation strain; *Y*_ind_, indentation yield strength; *σ*_ys_, uniaxial yield strength; *R*_G_, average grain size (radius).
